# Multidisciplinary clinic attendance and patterns of care in the U.S. National ALS Registry, 2013–2023

**DOI:** 10.1080/21678421.2026.2717968

**Published:** 2026-08-19

**Authors:** D. KEVIN HORTON, JAIME RAYMOND, THEODORE LARSON, STEPHEN A. GOUTMAN, PAUL MEHTA

**Affiliations:** 1 Agency for Toxic Substances and Disease Registry/Centers for Disease Control and Prevention, Atlanta, GA, USA; 2 Department of Neurology, University of Michigan, Ann Arbor, MI, USA; 3 Scott Pranger ALS Center, University of Michigan, Ann Arbor, MI, USA

**Keywords:** Amyotrophic lateral sclerosis, multidisciplinary care, clinical interventions, healthcare utilization, National ALS Registry

## Abstract

**Background:**

Amyotrophic lateral sclerosis (ALS) multidisciplinary clinics (MDCs) are the standard model of care, but national-level data on utilization and outcomes in the United States are lacking.

**Objective:**

To compare baseline characteristics and symptoms, clinical interventions and specialized care processes, and healthcare utilization among U.S. National ALS Registry (Registry) participants by MDC attendance.

**Methods:**

Registry data from 2013 to 2023 were analyzed for participants completing demographic and clinical surveys, stratified by MDC attendance (attendance vs. no attendance by survey completion).

**Results:**

Among 4764 participants, 77.0% reported attending an MDC. Baseline characteristics were similar between groups, including sex, age at diagnosis, and Amyotrophic Lateral Sclerosis Functional Rating Scale-Revised (ALSFRS-R) scores. MDC attendees reported lower prevalence of dysphagia (23.6% vs. 27.5%, *p* = 0.007) and bowel/bladder issues (10.7% vs. 14.3%, *p* = 0.001). Attendees were more likely to use evidence-based interventions, including wheelchairs/scooters, noninvasive ventilation, percutaneous endoscopic gastrostomy (PEG), communication devices, and ALS disease-modifying therapies, specifically riluzole (72.7% vs. 48.1%) and edaravone (10.5% vs. 2.6%) (all *p* < 0.001). MDC attendees also demonstrated higher rates of specialized care processes, including advance directive completion, genetic testing, and prior research participation (all *p* < 0.001). For healthcare utilization, MDC attendance was associated with fewer emergency department visits among those with any use (RR 0.90, 95% confidence interval (CI) 0.82–0.99) and shorter hospital stays (*β* = −2.67 days, 95% CI −4.56 to −0.78).

**Conclusion:**

MDC attendance was associated with greater uptake of evidence-based interventions, higher rates of specialized care processes, and reduced healthcare utilization intensity, consistent with previously reported multidisciplinary ALS care outcomes.

## Introduction

Amyotrophic lateral sclerosis (ALS) is a progressive neurodegenerative disease characterized by upper and lower motor neuron degeneration; median survival is 3–5 years from symptom onset, and disease-modifying therapies remain limited (1,2). In the United States (U.S.), approximately 35,000 individuals are living with ALS, with an estimated 5000 new cases diagnosed annually (3–5).

ALS multidisciplinary clinics (MDCs) are widely regarded as the standard model of care, integrating neurologists with allied health professionals to provide coordinated, symptom-directed management (6,7). MDC attendance has been associated in prior studies with improved patient outcomes, enhanced quality of life, and greater delivery of guideline-recommended interventions (8–12).

Despite these benefits, access to MDC care remains inconsistent across the U.S. (13), and national-level data on utilization and outcomes are lacking. Prior estimates are largely derived from regional or clinic-based cohorts (14), underscoring the need for large-scale, nationally representative data.

U.S. National ALS Registry data were used to compare clinical outcomes and care delivery processes among MDC attendees and non-attendees across three domains: baseline characteristics and symptoms, clinical interventions and specialized care processes, and healthcare utilization. This study provides the largest national evaluation of MDC attendance and real-world evidence on ALS care patterns in the U.S. to date.

## Methods

### Data source

The National ALS Registry (Registry), established by the Agency for Toxic Substances and Disease Registry (ATSDR) at the Centers for Disease Control and Prevention (CDC) in 2010, is a congressionally mandated program designed to better understand the epidemiology of ALS in the U.S. (15). The Registry combines administrative case ascertainment with patient-reported survey data to support national epidemiologic and clinical research. Because ALS is not a nationally notifiable disease in the U.S., Registry participants are identified through large administrative databases (e.g. Medicare) and a secure online self-enrollment portal (16–18). Participants may also complete up to 18 optional portal surveys capturing demographic, clinical, and exposure data (Supplementary Table S1).

### Study cohort and analysis period

Three Registry surveys provided data for the primary analyses: the Demographics survey, which provided demographic characteristics; the Disease Progression survey, which provided healthcare utilization measures (hospitalizations, emergency department (ED) visits, number of hospitalizations, number of ED visits, and days hospitalized) and Amyotrophic Lateral Sclerosis Functional Rating Scale-Revised (ALSFRS-R) assessments; and the Clinical and Care Utilization survey, which provided MDC attendance, symptoms, clinical interventions, specialized care processes, and site of onset information.

Participants were eligible if they completed the Demographics and Clinical and Care Utilization surveys, had at least one non-missing healthcare utilization measure, and reported their MDC attendance status at the time of survey completion. Participants who responded “don’t know” to the MDC attendance question were excluded. Survey responses were linked using a unique participant identifier common to all Registry surveys. The analysis period included survey responses collected between 2013 and 2023.

### Exposure definition

MDC attendance was dichotomized as “MDC attendance by survey completion” (reported current or previous attendance) or “No MDC attendance by survey completion” (reported no MDC attendance), with “don’t know” responses excluded. For readability, these groups are referred to throughout the remainder of the manuscript as “MDC attendees” and “MDC non-attendees,” respectively. Because the survey captured MDC attendance status at the time of survey completion, but did not collect information on the timing, frequency, or duration of MDC care, analyses were designed to evaluate associations between MDC attendance status at survey completion and participant characteristics, clinical care processes, and healthcare utilization.

### Outcomes

Outcomes were grouped into three domains reflecting both clinical measures and healthcare processes relevant to multidisciplinary ALS clinic care: baseline characteristics and symptoms, clinical interventions and specialized care processes, and healthcare utilization. Specialized care processes included advance directive completion, genetic testing, and research participation. Because MDC attendance was defined by status at survey completion, analyses were limited to cross-sectional associations. Accordingly, the association with survival could not be validly estimated using this exposure definition because classifying attendance status at survey completion onto the survival timescale would introduce temporal ambiguity and potential immortal-time/survivorship bias.

### Variables

All variables were derived from self-reported survey responses unless otherwise specified. Baseline characteristic variables included sex, age at diagnosis, geographic region, site of onset, and ALS Functional Rating Scale-Revised (ALSFRS-R) scores, a validated measure of functional status and disease severity in ALS (19). Geographic regions were assigned based on participant state of residence at enrollment and categorized into U.S. Census regions (Northeast, Midwest, South, and West). Site of onset was categorized as limb (arm/hand or leg/foot), bulbar (speech/swallowing muscles), trunk/global (neck, back, abdominal area, or total body weakness), or respiratory (breathing muscles) based on the initial body region with reported weakness before diagnosis. Baseline severity was approximated using the ALSFRS-R assessment closest to diagnosis within ±6 months, including retrospectively completed assessments. Symptom variables, including cramps/spasms, fasciculations, dysphagia, dysarthria, blood clots, bowel/bladder issues, pneumonia, and falls, reflected symptoms occurring after disease onset.

Clinical intervention variables included wheelchair or scooter use, noninvasive ventilation (NIV), tracheostomy, percutaneous endoscopic gastrostomy (PEG), communication device use (e.g. speech-generating devices), hospice enrollment, and use of U.S. Food and Drug Administration (FDA)-approved ALS therapies riluzole and edaravone. Specialized care process variables included advance directive completion, genetic testing, and research participation (prior and future).

Healthcare utilization variables included binary outcomes (any hospitalization or ED visit), count measures (number of hospitalizations and ED visits), and duration of hospitalization (days hospitalized).

### Statistical analysis

Baseline characteristics and symptoms were compared using chi-square tests for categorical variables and Welch’s *t*-tests for continuous variables. Time from diagnosis to Clinical and Care Utilization survey completion was non-normally distributed and therefore compared using the Wilcoxon rank-sum test; medians with interquartile ranges (IQRs) are reported. Edaravone analyses were restricted to 2018–2023, following its FDA approval in 2017 and incorporation into the Clinical and Care Utilization survey beginning in 2018.

Clinical interventions and specialized care processes were analyzed as categorical outcomes, with “don’t know” responses treated as missing and percentages calculated among non-missing responses; regression analyses were restricted to participants with complete outcome and covariate data.

Healthcare utilization was evaluated using logistic regression for binary outcomes, Poisson regression for count outcomes, and linear regression for continuous outcomes. Effect estimates are reported as odds ratios (ORs), rate ratios (RRs), and *β* coefficients with 95% confidence intervals (CIs). All models were adjusted for age and sex; logistic and Poisson models were additionally adjusted for site of onset. Models for days hospitalized were adjusted for age and sex only due to sparse data.

Sensitivity analyses were conducted to assess the potential influence of differential follow-up time. Healthcare utilization models additionally adjusted for time from diagnosis to Clinical and Care Utilization survey completion to address potential temporal bias; clinical intervention and specialized care process models further adjusted for age at diagnosis, sex, site of onset, and this same time variable, truncated at 60 months to reduce the influence of extreme values. Sensitivity analyses for clinical intervention and specialized care process outcomes are reported as adjusted odds ratios (AORs). Analyses were conducted using SAS version 9.4 (Cary, NC) (20).

### Ethics approval

The Registry operates under CDC Institutional Review Board approval, and all participants provide informed consent at registration.

## Results

From 2013 to 2023, 10,928 adults aged ≥18 years enrolled through the National ALS Registry online portal and completed at least one of the 18 surveys. Of these, 4764 participants met the study cohort criteria.

### Baseline characteristics and symptoms

Of the 4764 participants, 3667 (77.0%) reported attending an MDC by survey completion, whereas 1097 (23.0%) reported not attending an MDC by survey completion (Table 1). There were no significant differences in sex, age at diagnosis, site of onset, or baseline ALSFRS-R scores between groups. However, MDC attendance varied modestly by U.S. region (*p* = 0.015), with the lowest proportion of MDC attendees residing in Western states. MDC attendees also completed the Clinical and Care Utilization survey later after diagnosis than non-attendees (median time from diagnosis to survey completion: 6.1 [IQR 2.4–14.5] vs. 1.7 [IQR 0.6–6.4] months; *p* < 0.001). Symptom profiles were largely similar, though MDC attendees reported lower prevalence of dysphagia (23.6% vs. 27.5%, *p* = 0.007) and bowel or bladder issues (10.7% vs. 14.3%, *p* = 0.001).

### Clinical interventions and specialized care processes

MDC attendees were more likely to use evidence-based clinical interventions, including wheelchair or scooter (30.3% vs. 16.0%), NIV (33.3% vs. 15.9%), PEG (12.4% vs. 8.3%), communication devices (14.4% vs. 5.7%), riluzole (72.7% vs. 48.1%), and edaravone (10.5% vs. 2.6%) (all *p* < 0.001) (Table 2). There were no significant differences in tracheostomy use (1.6% vs. 1.5%) or hospice enrollment (4.1% vs. 4.3%) by attendance status. MDC attendees also demonstrated higher rates of specialized care processes, including advance directive completion (70.4% vs. 60.3%), genetic testing (34.5% vs. 18.9%), and prior research participation (25.3% vs. 7.3%) (all *p* < 0.001).

In sensitivity analyses (restricted to participants with complete covariate and timing data; *n* = 3555), associations between MDC attendance and clinical interventions and specialized care processes remained robust after adjustment for age, sex, site of onset, and time from diagnosis to Clinical and Care Utilization survey completion truncated at 60 months (Supplementary Table S2). The strongest associations were observed for prior research participation (AOR 4.28, 95% CI 3.20–5.72), edaravone use (AOR 3.60, 95% CI 2.20–5.90), and riluzole use (AOR 2.88, 95% CI 2.45–3.39).

### Healthcare utilization

MDC attendees had fewer ED visits among those reporting any ED use (RR 0.90, 95% CI 0.82–0.99; *p* = 0.03; *n* = 1489) and shorter hospital stays (6.52 vs. 9.16 days; *β* = −2.67, 95% CI −4.56 to −0.78; *p* = 0.006; *n* = 953) (Table 3). There were no significant differences in the likelihood of ED visits (OR 0.90, 95% CI 0.78–1.04; *p* = 0.14) or hospitalization (OR 0.98, 95% CI 0.83–1.16; *p* = 0.86), nor in the number of hospitalizations among those reporting any use (*n* = 953; RR 1.00, 95% CI 0.88–1.14; *p* = 0.99). In sensitivity analyses additionally adjusting for this truncated time variable, MDC attendance was associated with lower odds of any ED visit (AOR 0.71, 95% CI 0.60–0.84), while associations for ED visit frequency and hospitalization outcomes were otherwise materially unchanged (Supplementary Table S3).

## Discussion

In this U.S. National ALS Registry cohort, MDC attendance by survey completion was associated with greater use of evidence-based interventions, higher rates of specialized care processes, and reduced healthcare utilization intensity. These findings extend prior clinic-based and regional studies reporting associations between multidisciplinary care and improved ALS outcomes (7–12,21) and are consistent with National Academies recommendations emphasizing patient-centered care and quality-of-life outcomes in ALS (13).

Baseline characteristics, including sex, age at diagnosis, and site of onset, were largely similar between groups, consistent with prior U.S. Registry clinical findings (22) and the broader epidemiology of ALS. ALSFRS-R scores closest to diagnosis did not differ significantly between groups. Symptom patterns were generally comparable, though MDC attendees reported lower prevalence of dysphagia and bowel/bladder issues, which may reflect differences in disease trajectory or survey completion timing.

Clinical intervention differences were consistent with coordinated, guideline-concordant care, as MDC attendees had significantly higher use of wheelchairs/scooters, NIV, PEG, communication devices, and FDA-approved therapies (6,7), findings that remained materially similar in sensitivity analyses (Supplementary Table S2). Some differences may also reflect referral patterns whereby patients with greater supportive care needs are preferentially directed to MDC settings. Differences in disease-modifying therapy uptake were particularly notable: riluzole use was substantially higher among MDC attendees, highlighting greater uptake of guideline-recommended therapy (23–25), and the approximately fourfold difference in edaravone use suggests greater specialty access (26), though estimates should be interpreted cautiously given small numbers among non-attendees. Overall, MDC attendance was associated with broader uptake of guideline-recommended interventions in ALS care, although differences in functional status at the time these interventions were received cannot be excluded.

Advance directive completion, genetic testing, and prior research participation were all more common among MDC attendees, with similar results in sensitivity analyses (Supplementary Table S2). Greater advance directive completion was associated with MDC attendance and is consistent with earlier advance care planning, where early end-of-life discussions are widely recommended (6,7). Genetic testing rates were nearly twice as high among MDC attendees, which may reflect greater access to emerging gene-targeted treatments while also informing familial risk assessment. The more than threefold higher rate of prior research participation may similarly reflect greater access to clinical trials and other research opportunities. Together, these findings suggest that MDC attendance was associated with greater participation in specialized ALS care processes and research opportunities.

These findings may also provide additional context for previously reported benefits of multidisciplinary ALS care. Previous work has suggested that the survival benefit associated with multidisciplinary care is augmented by greater use of riluzole, NIV, and PEG, but that coordinated multidisciplinary care itself also contributes independently to improved outcomes (12). Our findings suggest that greater participation in specialized ALS care, including advance care planning, genetic testing, and research participation, may be one possible explanation for these observed associations. Patients receiving more comprehensive multidisciplinary care may receive earlier recognition of complications, more coordinated outpatient management, and improved anticipatory care, although these patterns cannot be distinguished from differences in referral pathways, healthcare access, disease course, or survey timing. Accordingly, causal relationships cannot be established in this observational study; however, these findings provide one possible explanation for the broader benefits of multidisciplinary ALS care reported previously.

Healthcare utilization patterns among MDC attendees included fewer ED visits and shorter hospital stays. Given that inpatient care is a major contributor to ALS costs (27), the observed reduction of approximately 2.7 hospitalized days, representing roughly a 29% reduction in mean days hospitalized, may translate into meaningful population-level cost savings. Shorter hospitalizations and fewer ED visits may also reduce logistical burden, caregiver strain, and disruption to outpatient care routines, outcomes that matter substantially in a disease where quality of life is a central goal of management. Sensitivity analyses yielded largely similar results overall, although the association for any ED visit strengthened after adjustment for time from diagnosis, while other utilization outcomes were materially unchanged (Supplementary Table S3). Collectively, the utilization patterns observed among MDC attendees are consistent with more proactive outpatient management and reduced intensity of acute healthcare use (28).

Structural and geographic barriers to MDC care remain important determinants of access, particularly for patients with bulbar-onset disease whose communication and travel difficulties limit engagement (13,29). The modestly lower MDC attendance observed in Western states is consistent with geographic dispersion and distance to specialized centers (30) and highlights where targeted outreach or infrastructure investment may be most needed. Although the survey cohort included a higher proportion of participants from the Midwest and a lower proportion from the Northeast than the overall National ALS Registry population previously reported (31), the South and West were similarly represented. These regional differences may limit the generalizability of our findings and could contribute to the observed regional variation in MDC attendance. The approximately 23% of Registry participants who had not attended an MDC at the time of survey completion also represent a group for whom barriers to MDC access and referral pathways warrant further study. Telehealth and hybrid care models may help improve access for these patients, particularly those with mobility or communication limitations (32–34). Addressing these structural barriers will be essential to ensuring that the benefits of MDC care reach all patients equitably.

This study has limitations. The observational design limits causal inference, and the Registry’s self-enrollment mechanism likely captures more healthcare-engaged participants, introducing selection and survivorship bias and contributing to heterogeneity across disease stages and healthcare settings. Residual confounding from geographic, socioeconomic, and healthcare access factors may further influence findings. MDC attendees completed the Clinical and Care Utilization survey later after diagnosis, potentially providing greater opportunity for clinical interventions, healthcare utilization events, and specialized care processes and research participation to occur or be reported, though sensitivity analyses yielded materially similar results. The study period also spanned a decade during which specialized ALS care evolved, including greater emphasis on advance care planning, genetic testing, and research participation, which may have influenced some observed care patterns. In addition, MDC attendance was modeled as a binary self-reported exposure without information on frequency, duration, or timing of care, precluding dose-response assessment and survival analyses. Some participants classified as not attending an MDC by survey completion may not yet have attended, or may have been unable to attend, an MDC by the time they completed the Clinical and Care Utilization survey. Referral patterns and healthcare-seeking behaviors may also have influenced MDC attendance. Finally, ALSFRS-R was used to approximate baseline severity without longitudinal follow-up, and variability in assessment timing may have reduced comparability across participants.

## Conclusion

In the largest national analysis of MDC attendance in the U.S. to date, MDC attendance was associated with greater uptake of evidence-based clinical interventions, higher rates of specialized care processes and research participation, and reduced healthcare utilization intensity. These findings provide additional evidence supporting efforts to expand MDC access in ALS care delivery, including through telehealth and hybrid models, and underscore the utility of large-scale registry data in identifying gaps and guiding equitable, patient-centered care.

## Supplementary Material

Supplementary Material

Supplemental data for this article can be accessed online at https://doi.org/10.1080/21678421.2026.2717968.

## Figures and Tables

**Table 1.** Baseline characteristics and symptoms among national ALS Registry participants by ALS multidisciplinary clinic attendance status, 2013–2023 (*N* = 4764)^a^.

Characteristic	No MDC attendance by survey completion (*n* = 1097, 23.0%) *n*, %	MDC attendance by survey completion (*n* = 3667, 77.0%) *n*, %	*p* Value^g^
Baseline			
Sex			0.78
Male	651 (59.3%)	2159 (58.9%)	
Female	446 (40.7%)	1508 (41.1%)	**0.015**
Geographic region^b^			
Northeast	135 (12.4%)	517 (14.1%)	
Midwest	333 (30.7%)	1138 (31.1%)	
South	344 (31.7%)	1241 (34.0%)	
West	273 (25.2%)	759 (20.8%)	
Age at diagnosis, years, mean (SD)^c^	60.8 (10.8)	60.2 (10.5)	0.09
Site of initial disease onset^d^			0.08
Limb	768 (70.0%)	2631 (71.7%)	
Arm or hand	365 (33.3%)	1271 (34.7%)	
Leg or foot	403 (36.7%)	1360 (37.1%)	
Bulbar (speech/swallowing muscles)	246 (22.4%)	799 (21.8%)	
Trunk/global	62 (5.7%)	146 (4.0%)	
Neck, back, or abdominal area	26 (2.4%)	81 (2.2%)	
Total body	36 (3.3%)	65 (1.8%)	
Respiratory	20 (1.8%)	85 (2.3%)	
ALSFRS-R score (closest to diagnosis), mean (SD)^e^	36.5 (7.0)	36.6 (7.0)	0.85
Time from diagnosis to Clinical and Care Utilization survey completion, months, median (IQR)	1.7 (0.6–6.4)	6.1 (2.4–14.5)	**<0.001**
Symptoms^f^			
Cramps/spasms	638 (58.2%)	2023 (55.2%)	0.08
Fasciculations (twitching)	619 (56.4%)	1981 (54.0%)	0.16
Dysphagia (swallowing difficulty)	302 (27.5%)	864 (23.6%)	**0.007**
Dysarthria (speech difficulty)	397 (36.2%)	1229 (33.5%)	0.10
Blood clot(s)	37 (3.4%)	131 (3.6%)	0.75
Bowel/bladder issues	157 (14.3%)	393 (10.7%)	**0.001**
Pneumonia	54 (4.9%)	195 (5.3%)	0.60
Falls	229 (20.9%)	843 (23.0%)	0.14

a Missing values were excluded from statistical comparisons. Subcategory percentages may not sum to parent category totals due to rounding.

b Region categories were defined using U.S. Census regions based on participant state of residence at enrollment. Participants with missing state information were excluded from regional analyses.

c SD: standard deviation; IQR: interquartile range.

d Site of onset data were missing for one participant in the no MDC attendance by survey completion group and six in the MDC attendance by survey completion group; percentages are based on total group denominators.

e ALSFRS-R values represent the validated 48-point score closest to diagnosis within ±6 months; means were calculated among 2584 participants with an available assessment.

f Symptoms are based on self-reported responses and indicate whether participants reported each symptom (yes/no).

g *p* Values for categorical variables were calculated using chi-square tests and continuous variables using Welch’s *t*-test. Time from diagnosis to Clinical and Care Utilization survey completion was compared using the Wilcoxon rank-sum test and is reported as median (IQR). Bold *p* values indicate statistical significance.

**Table 2.** Clinical interventions and specialized care processes among national ALS Registry participants by ALS multidisciplinary clinic attendance status, 2013–2023^a,b^.

Characteristic	No MDC attendance by survey completion (*n* = 1097, 23.0%) *n*, %	MDC attendance by survey completion (*n* = 3667, 77.0%) *n*, %	*p* Value^e^
Clinical interventions			
Wheelchair or scooter	176 (16.0%)	1110 (30.3%)	**<0.001**
Noninvasive ventilation (NIV)	174 (15.9%)	1221 (33.3%)	**<0.001**
Tracheostomy	16 (1.5%)	58 (1.6%)	0.77
Percutaneous endoscopic gastrostomy (PEG)	91 (8.3%)	453 (12.4%)	**<0.001**
Communication device	63 (5.7%)	528 (14.4%)	**<0.001**
Hospice enrollment	47 (4.3%)	152 (4.1%)	0.84
Riluzole use^c^	528 (48.1%)	2666 (72.7%)	**<0.001**
Edaravone use^c^	28 (2.6%)	385 (10.5%)	**<0.001**
Specialized care processes			
Advance directive completion	661 (60.3%)	2583 (70.4%)	**<0.001**
Genetic testing	207 (18.9%)	1266 (34.5%)	**<0.001**
Research participation (prior)	80 (7.3%)	929 (25.3%)	**<0.001**
Interested in participating in research (future)^d^	902 (82.2%)	3088 (84.2%)	0.16

a Missing values were excluded from statistical comparisons. Percentages are based on non-missing responses; denominators vary across variables due to item-level missing data.

b Clinical intervention and specialized care process variables were derived from self-reported survey responses.

c FDA-approved ALS medications reflect current or prior use; edaravone analyses were restricted to 2018–2023 following FDA approval in 2017.

d Future research participation reflects interest or willingness to participate, rather than prior behavior; “don’t know” responses were classified as missing.

e *p* Values were calculated using chi-square tests and were not adjusted for multiple comparisons. Bold *p* values indicate statistical significance.

**Table 3.** Healthcare utilization outcomes among national ALS Registry participants by ALS multidisciplinary clinic attendance status, 2013–2023^a,b,c,d^.

Outcome	No MDC attendance by survey completion	MDC attendance by survey completion	Effect	95% CI	*p* Value
Any hospitalization, *n/N* (%)	222/1097 (20.2%)	731/3667 (19.9%)	OR 0.98	0.83–1.16	0.86
Any emergency department visit, *n/N* (%)	363/1097 (33.1%)	1126/3667 (30.7%)	OR 0.90	0.78–1.04	0.14
Number of hospitalizations per participant, mean (SD)	1.38 (0.82)	1.38 (0.92)	RR 1.00	0.88–1.14	0.99
Number of ED visits per participant, mean (SD)	1.60 (1.21)	1.44 (0.84)	RR 0.90	0.82–0.99	**0.03**
Hospital days per participant, mean (SD)	9.16 (14.35)	6.52 (11.94)	*β* = −2.67	−4.56 to −0.78	**0.006**

a *n/N*: number with outcome/number assessed; SD: standard deviation; ED: emergency department; OR: odds ratio; RR: rate ratio; *β*: linear regression coefficient; CI: confidence interval.

b Descriptive counts and percentages are based on the full analytic cohort. Missing values were excluded from statistical comparisons; adjusted analyses included only participants with complete outcome and covariate data. Healthcare utilization variables were derived from harmonized participant-level Disease Progression survey responses.

c Models were adjusted for age and sex; logistic and Poisson models were additionally adjusted for site of onset. Models for days hospitalized were adjusted for age and sex only because of sparse data.

d Analyses of the number of hospitalizations, the number of emergency department visits, and hospital days were restricted to participants who reported the corresponding event and provided nonmissing information for that outcome. The analytic sample sizes were 953 participants for the hospitalization-count analysis, 1489 participants for the emergency department visit-count analysis, and 953 participants for the hospital-days analysis. These analytic sample sizes represent the number of participants included in each analysis and should not be interpreted as the total numbers of hospitalizations, emergency department visits, or hospital days.

Bold p-values indicate statistical significance (p < 0.05).

## Data Availability

The data supporting the findings of this study are not publicly available due to the privacy risks of the subjects and the policies of the data providers.

## References

[1] Feldman EL, Goutman SA, Petri S, Mazzini L, Savelieff MG, Shaw PJ, Amyotrophic lateral sclerosis. Lancet. 2022;400:1363–80. 36116464 10.1016/S0140-6736(22)01272-7 PMC10089700

[2] Quigley SE, Quigg KH, Goutman SA. Genetic and mechanistic insights inform amyotrophic lateral sclerosis treatment and symptomatic management: current and emerging therapeutics and clinical trial design considerations. CNS Drugs. 2025;39:949–93. 40897992 10.1007/s40263-025-01217-0 PMC12423166

[3] Mehta P, Raymond J, Nair T, Han M, Berry J, Punjani R, Amyotrophic lateral sclerosis estimated prevalence cases from 2022 to 2030, data from the National ALS Registry. Amyotroph Lateral Scler Frontotemporal Degener. 2025;26:290–5. 39749668 10.1080/21678421.2024.2447919

[4] Mehta P, Raymond J, Punjani R, Larson T, Han M, Bove F, Incidence of amyotrophic lateral sclerosis in the United States, 2014–2016. Amyotroph Lateral Scler Frontotemporal Degener. 2022;23:378–82. 35023792 10.1080/21678421.2021.2023190

[5] Wagner L, Rechtman L, Jordan H, Ritsick M, Sanchez M, Sorenson E, State and metropolitan area-based amyotrophic lateral sclerosis (ALS) surveillance. Amyotroph Lateral Scler Frontotemporal Degener. 2015; 17:128–34. 26399278 10.3109/21678421.2015.1074699 PMC4732418

[6] Miller RG, Jackson CE, Kasarskis EJ, England JD, Forshew D, Johnston W, Practice parameter update: the care of the patient with amyotrophic lateral sclerosis: multidisciplinary care, symptom management, and cognitive/behavioral impairment. Neurology. 2009;73: 1227–33. 19822873 10.1212/WNL.0b013e3181bc01a4 PMC2764728

[7] Van Damme P, Al-Chalabi A, Andersen PM, Chiò A, Couratier P, De Carvalho M, European Academy of Neurology (EAN) guideline on the management of amyotrophic lateral sclerosis in collaboration with European Reference Network for Neuromuscular Diseases (ERN EURO-NMD). Eur J Neurol. 2024;31:e16264. 38470068 10.1111/ene.16264 PMC11235832

[8] Van den Berg JP, Kalmijn S, Lindeman E, Veldink JH, de Visser M, Van der Graaff MM, Multidisciplinary ALS care improves quality of life in patients with ALS. Neurology. 2005;65:1264–7. 16247055 10.1212/01.wnl.0000180717.29273.12

[9] Hogden A, Foley G, Henderson RD, James N, Aoun SM. Amyotrophic lateral sclerosis: improving care with a multidisciplinary approach. J Multidiscip Healthc. 2017; 10:205–15. 28579792 10.2147/JMDH.S134992 PMC5446964

[10] Traynor BJ, Alexander M, Corr B, Frost E, Hardiman O. Effect of a multidisciplinary ALS clinic on ALS survival: a population based study, 1996–2000. J Neurol Neurosurg Psychiatry. 2003;74:1258–61. 12933930 10.1136/jnnp.74.9.1258 PMC1738639

[11] Rooney J, Byrne S, Heverin M, Tobin K, Dick A, Donaghy C, A multidisciplinary clinic approach improves survival in ALS: a comparative study of ALS in Ireland and Northern Ireland. J Neurol Neurosurg Psychiatry. 2015;86:496–501. 25550416 10.1136/jnnp-2014-309601

[12] Aridegbe T, Kandler R, Walters SJ, Walsh T, Shaw PJ, McDermott CJ. The natural history of motor neuron disease: assessing the impact of specialist care. Amyotroph Lateral Scler Frontotemporal Degener. 2013;14:13–9. 22642305 10.3109/17482968.2012.690419

[13] National Academies of Sciences, Engineering, and Medicine. Living with ALS. Washington, DC: National Academies Press; 2024.

[14] Stephens HE, Young J, Felgoise SH, Simmons Z. Multidisciplinary ALS clinics in the USA: a comparison of those who attend and those who do not. Amyotroph Lateral Scler Frontotemporal Degener. 2015;16:196–201. 25602166 10.3109/21678421.2014.994530

[15] U.S. Public Health Service. ALS Registry Act. Public Law 110-373. Washington, DC; 2008.

[16] Horton DK, Mehta P, Antao VC. Quantifying a nonnotifiable disease in the US: the National ALS Registry model. JAMA. 2014;312:1097–8. 25057819 10.1001/jama.2014.9799 PMC4550214

[17] Mehta P, Raymond J, Zhang Y, Yoon H, Bede P, Genge A, Prevalence of ALS in the US, 2018. Amyotroph Lateral Scler Frontotemporal Degener. 2023;24:702–8. 37602649 10.1080/21678421.2023.2245858

[18] Antao VC, Horton DK. The National ALS Registry. J Environ Health. 2012;75:28–30. PMC4549968 22866401

[19] Cedarbaum JM, Stambler N, Malta E, Fuller C, Hilt D, Thurmond B, The ALSFRS-R: a revised ALS functional rating scale. J Neurol Sci. 1999;169:13–21. 10540002 10.1016/s0022-510x(99)00210-5

[20] SAS Institute Inc. SAS software, version 9.4. Cary, NC: SAS Institute Inc.; 2013.

[21] Chiò A, Bottacchi E, Buffa C, Mutani R, Mora G, PARALS. Positive effects of tertiary centres for amyotrophic lateral sclerosis on outcome and use of hospital facilities. J Neurol Neurosurg Psychiatry. 2006;77: 948–50. 16614011 10.1136/jnnp.2005.083402 PMC2077622

[22] Raymond J, Oskarsson B, Mehta P, Horton K. Clinical characteristics of a large cohort of US participants enrolled in the National Amyotrophic Lateral Sclerosis (ALS) Registry, 2010–2015. Amyotroph Lateral Scler Frontotemporal Degener. 2019;20:413–20. 31131638 10.1080/21678421.2019.1612435 PMC6946020

[23] Bensimon G, Lacomblez L, Meininger V, ALS/Riluzole Study Group. A controlled trial of riluzole in ALS. N Engl J Med. 1994;330:585–91. 8302340 10.1056/NEJM199403033300901

[24] Lacomblez L, Bensimon G, Leigh PN, Guillet P, Meininger V. Dose-ranging study of riluzole in ALS. ALS/Riluzole Study Group-II. Lancet. 1996;347:1425–31. 8676624 10.1016/s0140-6736(96)91680-3

[25] Miller RG, Mitchell JD, Moore DH. Riluzole for amyotrophic lateral sclerosis (ALS)/motor neuron disease (MND). Cochrane Database Syst Rev. 2012;2012:CD001447. 11687111 10.1002/14651858.CD001447

[26] Writing Group; Edaravone (MCI-186) ALS 19 Study Group. Safety and efficacy of edaravone in well defined patients with amyotrophic lateral sclerosis: a randomised, double-blind, placebo-controlled trial. Lancet Neurol. 2017;16:505–12. 28522181 10.1016/S1474-4422(17)30115-1

[27] Berry JD, Blanchard M, Bonar K, Drane E, Murton M, Ploug U, Epidemiology and economic burden of amyotrophic lateral sclerosis in the US: a literature review. Amyotroph Lateral Scler Frontotemporal Degener. 2023; 24:436–48. 36748473 10.1080/21678421.2023.2165947

[28] Sugisawa T, Morioka H, Hirayama T, Kano O, Ebihara S. Multidisciplinary clinic contributes to the decreasing trend in the number of emergency hospitalizations for amyotrophic lateral sclerosis in Japan. J Clin Neurosci. 2023;107:133–7. 36565495 10.1016/j.jocn.2022.10.011

[29] Galvin M, Madden C, Maguire S, Heverin M, Vajda A, Staines A, Patient journey to a specialist ALS multidisciplinary clinic. BMC Health Serv Res. 2015;15:571. 26700026 10.1186/s12913-015-1229-x PMC4690216

[30] Horton DK, Graham S, Punjani R, McTigue M, Kliethermes SJ, Sloop A, Spatial analysis of ALS cases and proximity to clinics. Amyotroph Lateral Scler Frontotemporal Degener. 2018;19:126–33. 29262737 10.1080/21678421.2017.1406953 PMC5815888

[31] Mehta P, Kaye W, Raymond J, Punjani R, Larson T, Cohen J, Prevalence of amyotrophic lateral sclerosis—US, 2015. MMWR Morb Mortal Wkly Rep. 2018;67:1285–9. 30462626 10.15585/mmwr.mm6746a1 PMC6289079

[32] Haulman A, Geronimo A, Chahwala A, Simmons Z. The use of telehealth to enhance care in ALS and other neuromuscular disorders. Muscle Nerve. 2020;61:682–91. 32297678 10.1002/mus.26838 PMC10797583

[33] Van De Rijn M, Paganoni S, Levine-Weinberg M, Campbell K, Swartz Ellrodt A, Estrada J, Telemedicine in a multidisciplinary ALS clinic. Amyotroph Lateral Scler Frontotemporal Degener. 2018; 19:143–8. 29250986 10.1080/21678421.2017.1392577

[34] Helleman J, van Eijk RPA, Beelen A, van den Berg LH, Wokke JHJ, van Damme P, Telemedicine for multidisciplinary ALS care. Amyotroph Lateral Scler Frontotemporal Degener. 2020;21:404–11.

