# Supplementary material for: Multidisciplinary clinic attendance and patterns of care in the U.S. National ALS Registry, 2013–2023
Source: Amyotroph Lateral Scler Frontotemporal Degener. Author manuscript; Available in PMC 2026 Sep 14. (PMC13574221; doi:10.1080/21678421.2026.2717968)
Supplement: Supplementary file 1 [file NIHMS2206683-supplement-Supplementary_Material.docx]

**Supplementary Material**

**Supplementary Table S1: List of current surveys in the National ALS Registry web portal and release dates**

| **Surveys** | **Type** | **Active dates**^a^ |
| --- | --- | --- |
| 1 | Demographics | Oct 2010–2025 |
| 2 | Occupation | Oct 2010–2025 |
| 3 | Military History | Oct 2010–2025 |
| 4 | Smoking and Alcohol | Oct 2010–2025 |
| 5 | Physical Activity | Oct 2010–2025 |
| 6 | Family History of ALS | Oct 2010–2025 |
| 7 | Disease Progression | Oct 2010–2025 |
| 8 | Lifetime Residential History | May 2014–2025 |
| 9 | Lifetime Occupational History | May 2014–2025 |
| 10 | Residential Pesticide Use | May 2014–2025 |
| 11 | Hobbies with Toxicant Exposure | Aug 2014–2025 |
| 12 | Reproductive History Women | Aug 2014–2025 |
| 13 | Caffeine Consumption | Aug 2014–2025 |
| 14 | Head and Neck Injuries | Dec 2014–2025 |
| 15 | Health Insurance Status | Dec 2014–2025 |
| 16 | Open Ended Etiologic Questions | Nov 2013–2025 |
| 17 | Clinical and Care Utilization | Nov 2013–2025 |
| 18 | Sports Survey | Jan 2021–2025 |

^a^Active dates reflect the full operational period of each survey. All surveys are completed once, except the Disease Progression survey, which is longitudinal and may be completed repeatedly to capture serial Amyotrophic Lateral Sclerosis Functional Rating Scale–Revised (ALSFRS-R) assessments and disease progression over time.

**Supplementary Table S2. Sensitivity analyses of clinical interventions and specialized care processes associated with ALS multidisciplinary clinic attendance among National ALS Registry participants, 2013–2023**

| **Outcome**^a^ | **AOR**^c^ | **95% CI** |
| --- | --- | --- |
| **Clinical Interventions** | | |
| Wheelchair or scooter | 1.89 | 1.51–2.37 |
| Noninvasive ventilation (NIV) | 2.54 | 2.05–3.15 |
| Percutaneous endoscopic gastrostomy (PEG) | 1.50 | 1.03–2.18 |
| Communication device | 2.85 | 2.02–4.01 |
| Riluzole use | 2.88 | 2.45–3.39 |
| Edaravone use^b^ | 3.60 | 2.20–5.90 |
| **Specialized Care Processes** | | |
| Advance directives | 1.46 | 1.23–1.74 |
| Genetic testing | 2.23 | 1.83–2.72 |
| Research participation (prior) | 4.28 | 3.20–5.72 |

^a^Outcomes without statistically significant associations in primary analyses (tracheostomy, hospice enrollment, and future research interest) are not shown.

^b^Restricted to participants with edaravone-era data availability (2018–2023).

^c^AOR, adjusted odds ratios. Adjusted for age at diagnosis, sex, site of onset, and time from diagnosis to Clinical and Care Utilization survey completion truncated at 60 months to reduce the influence of extreme values. Sensitivity analyses were restricted to participants with complete covariate and timing data (n = 3,555).

**Supplementary Table S3. Sensitivity analyses of healthcare utilization outcomes by ALS multidisciplinary clinic attendance status among National ALS Registry participants, 2013–2023**^a^

| **Outcome**^b^ | **Primary model**^c^ **effect** | **95% CI** | **Sensitivity model**^d^ **effect** | **95% CI** | **Interpretation** |
| --- | --- | --- | --- | --- | --- |
| Any hospitalization | OR 0.98 | 0.83–1.16 | AOR 0.86 | 0.69–1.06 | Materially similar |
| Any emergency department visit | OR 0.90 | 0.78–1.04 | AOR 0.71 | 0.60–0.84 | Association strengthened after adjustment |
| Number of hospitalizations per participant | RR 1.00 | 0.88–1.14 | RR 1.00 | 0.85–1.18 | Materially similar |
| Number of ED visits per participant | RR 0.90 | 0.82–0.99 | RR 0.92 | 0.82–1.04 | Materially similar |
| Hospital days per participant | β = −2.67 | −4.56 to −0.78 | β = −2.90 | −5.25 to −0.56 | Materially similar |

^a^Abbreviations: OR, odds ratio; AOR, adjusted odds ratio; RR, rate ratio; β, linear regression coefficient; CI, confidence interval; ED, emergency department.

^b^Sample sizes varied by outcome and were smaller than those in Table 3 because sensitivity models required complete outcome, covariate, and timing data: any hospitalization, n = 4,444; any emergency department visit, n = 4,411; number of hospitalizations per participant, n = 625; number of ED visits per participant, n = 1,058; hospital days per participant, n = 665.

^c^Primary model effects correspond to the adjusted estimates presented in Table 3.

^d^Sensitivity models additionally adjusted for capped time from diagnosis to Clinical and Care Utilization survey completion and included adjustment for age, sex, and site of onset (where applicable). Analyses were restricted to participants with complete outcome, covariate, and timing data.
